# Nonoperative Management May Be a Viable Approach to Plexiform Neurofibroma of the Porta Hepatis in Patients with Neurofibromatosis-1

**DOI:** 10.1155/2018/7814763

**Published:** 2018-04-15

**Authors:** Natesh Yepuri, Rana Naous, Camille Richards, Dilip Kittur, Ajay Jain, Mashaal Dhir

**Affiliations:** ^1^Department of Surgery, SUNY Upstate Medical University, Syracuse, NY 13210, USA; ^2^Department of Pathology, SUNY Upstate Medical University, Syracuse, NY 13210, USA

## Abstract

**Background:**

Plexiform neurofibroma (PNF) in the porta hepatis (PH) is an unusual manifestation of neurofibromatosis-1 (NF-1). Resection is often recommended given the risk of malignant transformation. We encountered a challenging case in clinical practice which prompted us to report our findings and perform a systematic review on the management of these tumors.

**Methods:**

We reported the case of a 31-year-old woman with NF-1 and PNF of the PH. PRISMA 2009 guidelines were followed for systematic review.

**Results:**

Our patient was found to have unresectable disease at exploration. After >5 years of follow-up, she continued to have stable disease on imaging. We identified 12 studies/case reports including 10 adult and 6 pediatric patients with PNF of PH. None of the 7 adult patients with NF-1 and PNF of PH underwent a successful tumor resection. All pediatric patients were managed with surveillance alone. All but one pediatric patient had NF-1. None of the reported cases of PNF of PH had malignant transformation.

**Conclusion:**

Our findings suggest that PNFs of PH in the setting of NF-1 are often unresectable and may have an indolent course. Surveillance alone may be a reasonable option in some patients; however, further studies are needed.

## 1. Introduction

Neurofibromatosis-1 (NF-1) is a progressive multisystem neurocutaneous genetic disorder with an autosomal dominant inheritance [1]. NF-1 is caused by mutations in the NF-1 gene and affects both genders equally, with an incidence of one in 2500–3000 births [1–3]. NF-1 gene mutations can lead to dysregulation of RAS/MAP kinase and mammalian target of rapamycin (mTOR) signaling pathways which can lead to development of several types of neoplasms [1, 2]. Almost half of these mutations occur de novo in patients with no family history [1, 2] and there are several genotype-phenotype variations [1, 2]. Given the complex underlying genetics, diagnosis is often based on clinical features such as café-au-lait spots, Lisch nodules (iris hamartomas), and neurofibromas [1–3]. Neurofibromas are one of the most common and characteristic clinical manifestations of NF-1. These tumors can be located superficially in the skin or internally in the entire body including mediastinum, retroperitoneum, or GI tract. Plexiform neurofibromas (PNFs) are noncutaneous neurofibromas which are pathognomonic of NF-1 and overall one of the most challenging neoplasms to manage in NF-1.

PNFs are usually slow growing and affect 15–30% patients with NF-1. Clinical presentation of PNF is variable based on the organ of involvement, that is, mediastinum, retroperitoneum or GI tract, and so on [4]. In contrast to cutaneous neurofibromas which grow intraneurally, PNFs can involve an entire plexus of nerves and demonstrate an infiltrative pattern. Although PNFs are benign they have the potential to transform into malignant peripheral nerve sheath tumors which demonstrate aggressive behavior. Many experts recommend resection given the underlying malignant potential. However, locally infiltrative pattern can make these resections quite difficult.

We encountered a challenging case of PNF of Porta hepatis (PH) in clinical practice which prompted us to report our findings. PNFs of the PH are extremely rare and even more technically challenging to resect given the location and close relationship with the biliary and vascular inflow to the liver. There is no consensus on the management of these tumors given the rarity of clinical presentation. We hereby present the findings of our case. Additionally, we performed a systematic review of the literature to summarize the current evidence on the management of PNFs involving the PH.

## 2. Methodology

### 2.1. Systematic Review: Plexiform Neurofibroma of Porta Hepatis

**Figure 1:**
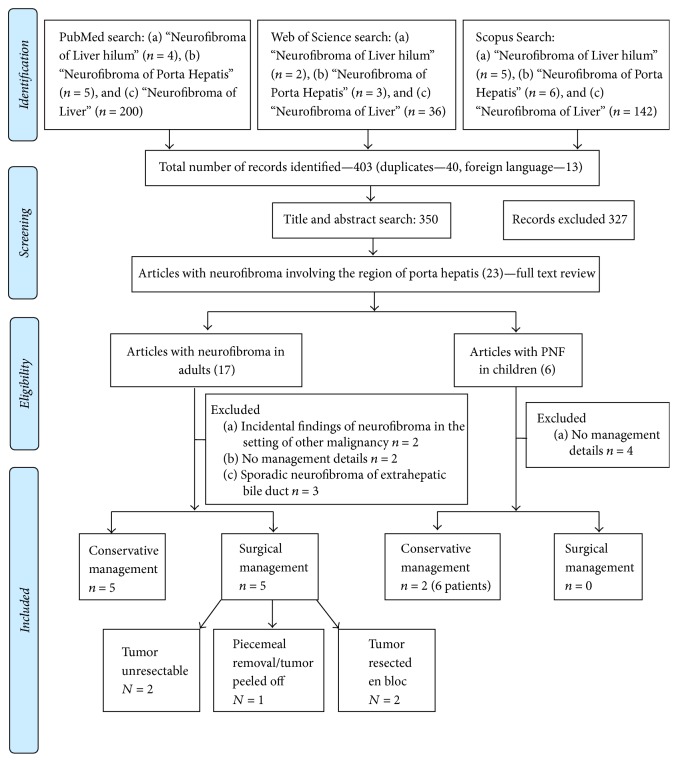
Study flow diagram and selection strategy.

A literature search was performed using the PubMed, Scopus, and Web of Science databases. Final search was conducted in 10/2017. Following search criteria were utilized: (a) “Neurofibroma of Liver hilum,” (b) “Neurofibroma of Porta Hepatis,” and (c) “Neurofibroma of Liver.” For data extraction, first author (N. Y.) and senior author (M. D.) selected the studies and assessed for eligibility. A total of 403 articles were identified. Duplicates (*n* = 40) and articles in foreign languages (*n* = 13) were excluded. Title and abstract review were conducted for the remaining 350 articles. Inclusion criteria included case reports/series of neurofibroma in the region of porta hepatis with intent to include only PNF in the region of PH in the final qualitative synthesis. Full texts were reviewed for 23 articles. A backward search was also performed using cross references from the bibliographies of relevant articles and review articles to ensure a comprehensive search. Articles discussing imaging findings only without clinical management were excluded. Nineteen studies were included in the final qualitative synthesis. Studies reporting on adults (Age ≥ 18 years) and children (<18 years) were summarized separately.  Figure 1 summarizes the search strategy and inclusion/exclusion criteria per the PRISMA 2009 guidelines for systematic reviews.

## 3. Results

### 3.1. Case Presentation

**Figure 2:**
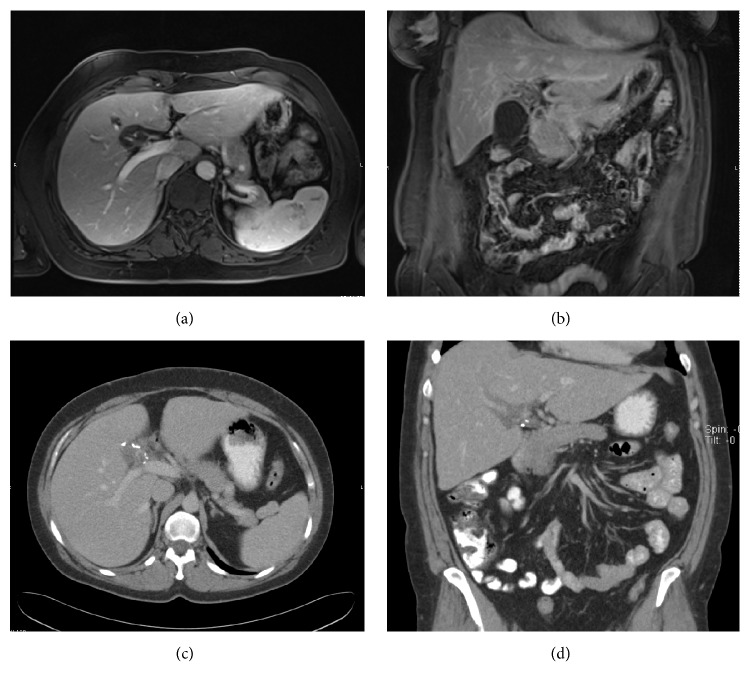
(a and b) Representative axial and coronal images from contrast enhanced preoperative MRI of the patient. Encasement of hepatic artery with extension of the mass predominantly towards the right side is noted in GB fossa. (c & d) Follow-up postoperative CT scans 5 years later depicting almost stable appearance of the mass.

A 31-year-old woman previously diagnosed with NF-1 presented to the emergency department with RUQ pain. Physical examination, liver function tests, and blood chemistries were unremarkable. Abdominal ultrasound (US) revealed a lobulated hypoechoic mass in the gallbladder fossa. A subsequent MRI scan noted a T1 hypointense and heterogeneously T2 hyperintense mass encasing the hepatic artery and portal vein within the PH (Figures 2(a) and 2(b)). There was no loss of signal on the out-of-phase images. The preoperative diagnosis for this mass was felt to be a neurofibroma. Given the symptomatic nature of the mass, decision was made to proceed with resection.

**Figure 3:**
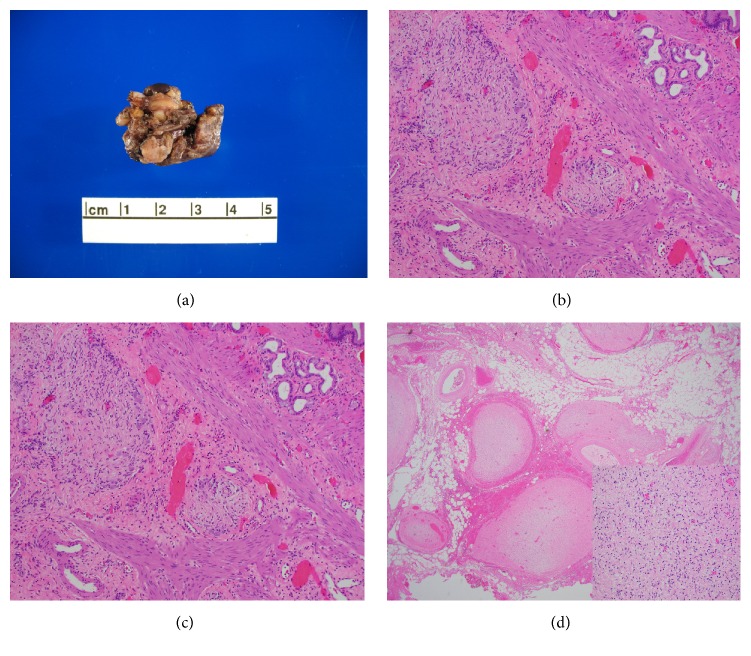
(a) Gross morphology of “Mass in Gallbladder.” Note the lobulated and nodular overall surface resembling a “bag of worms.” The mass measured in total 3.2 × 2.2 × 1.3 cm. (b) Plexiform neurofibroma involving the muscularis propria of the gallbladder wall (H&E, 200x). (c) Higher magnification highlights the loosely arranged spindle shaped cells of plexiform neurofibroma with peripheral entrapment of native ganglion cells (H&E, 400x). (d) Plexiform neurofibroma residing within the fibrofatty tissue adjacent to the gallbladder. (H&E, 200x) (inset) plexiform neurofibroma showing typical histologic findings with loosely arranged comma-shaped nuclei in a myxoid stroma (H&E, 600x).

During laparotomy a cholecystectomy was performed as the gallbladder was closely adherent to the mass. Further dissection revealed that mass was infiltrating the entire PH. There was intrahepatic extension along the right posterior, right anterior, and left portal pedicles. Given the intraoperative extent of the disease it was decided to abort surgical resection. Surgical pathology revealed plexiform neurofibroma involving the gallbladder without any evidence of malignant transformation.  Figure 3 highlights the gross and histopathologic features of PNF.

The patient's postoperative course was uneventful; however, the patient had neuropathic pain which was successfully managed with celiac plexus block and oral pain medications. The patient has been followed with serial MRIs and more recently with yearly CT scans of abdomen and pelvis. Over a period of six years the mass has remained stable.  Figure 2 shows the preoperative and postoperative representative images of the PNF along with gross and histopathologic images. Our patient has one of the longest reported follow-ups for PNF of the PH.

### 3.2. Systematic Review

We identified twenty-three studies/case reports on neurofibroma involving the region of PH. Seventeen studies included adult patients whereas six studies were on pediatric patients. Among the seventeen studies with adult patients 3 studies/cases were excluded as they reported on sporadic neurofibroma involving the bile ducts [5–7]. All three of these patients underwent successful resection of the sporadic neurofibroma. Two required roux-en-Y hepaticojejunostomy and one underwent a pancreaticoduodenectomy. Two studies reported on cases with incidentally found nonplexiform hepatic neurofibromas in the setting of other malignancies such as angiosarcoma and cholangiocarcinoma [8, 9]. These studies were excluded. Two other studies were excluded as one discussed imaging findings only and the other did not provide details on the management of the case [10, 11].

Ten adult patients with PNF of the PH were identified (Table 1) [4, 12–20]. Seven patients had NF-1 [4, 12, 14, 15, 17, 19, 20], 2 had PNF without NF-1, and status of NF-1 was unknown in one patient [13, 16, 18]. Five out of 10 patients underwent conservative management [4, 12, 13, 19, 20]. Resection was attempted in other 5 but only 2 underwent successful en bloc resection [16, 18]. Tumor was removed piecemeal in one patient [15] and found to be unresectable in the other two [14, 17]. Among the 7 patients with PNF of PH and NF-1, resection was not attempted in 4 due to imaging features suggestive of unresectability. Among the remaining 3 patients, tumor was found to be unresectable at exploration in two [14, 17] and could only be removed piecemeal in one patient [15].

We identified 6 articles with PNF of PH in patients < 18 years of age [21–26]. Four studies did not provide management details and were excluded [23–26]. Scheurkogel et al. reported a case of healthy 9-year-old male who underwent a renal ultrasound for intermittent low back pain and was found to have a periportal mass [21]. CT and MRI confirmed a mass in the PH extending into the liver and along the celiac axis. Probable diagnosis was PNF but given absence of family history and other clinical features of NF-1 diagnosis could not be confirmed without a biopsy. An open biopsy was then performed which confirmed PNF without transformation on final pathology (Figure 2). This PNF was thought to be unresectable on imaging and conservative management was appropriately chosen [21]. The largest series of PNF involving PH was reported by Delgado et al. [22]. PNF involving the PH was noted in 5/161 (3.1%) patients with NF-1. These authors suggested that periportal infiltration was the hallmark of PNF involving the liver. Imaging features of pediatric PNF are similar to those in adults. Age of patients varied from 4.1 to 17.8 years with a follow-up of 3 months–8.8 years. All patients were managed conservatively and underwent surveillance as tumors were very extensive and there was no evidence of transformation on MRI [22].

## 4. Discussion

Abdominal involvement in NF-1 occurs in the form of sporadic neurofibromas versus PNFs which can involve the liver [17, 24], mesentery [25, 27, 28], retroperitoneum [29], and gastrointestinal (GI) tract [30]. The GI involvement occurs in 10–25% of patients with NF-1 presenting as solitary or multiple neurofibromas, leiomyomas, and rarely PNF [30]. In the GI tract, neurofibromas are most commonly located in the ileum, followed by the jejunum, duodenum, and stomach [30]. Hepatic neurofibroma is a rare entity often associated with extensive abdominal and retroperitoneal involvement. It was reported that the prevalence of intrahepatic lesions was 2.3% of all PNFs involving the abdomen and pelvis [18]. Only a few cases of patients with intra-abdominal PNFs have been reported in the literature [22, 31, 32]. We encountered a challenging case of PNF involving the PH in the clinic and this prompted us to report our findings and review the literature on this rare but complex clinical entity.

PNFs are nonencapsulated tumors which have an interdigitating pattern of growth and can involve the entire plexus [32]. Histologically, neurofibromas are characterized by Schwann cells, perineural-like cells, and fibroblasts, with ovoid-to-spindle-shaped nuclei [31]. PNFs of the PH are extremely rare in incidence [4, 6, 18, 22, 24–26]. Clinical symptoms of PNF at PH are caused by compression of nerves derived from the left vagal trunk and sympathetic plexus causing visceral pain and ductal obstruction [22]. Most often these lesions are discovered incidentally during workup of vague abdominal symptoms. Imaging plays a key role in diagnosis of these tumors. On CT imaging, PNFs appear as multilobulated low-attenuation masses within a major nerve distribution [33]. This low attenuation is due to myxoid and mucinous stroma within these tumors [34]. On MRI, these tumors appear hypointense on T1 weighted images and heterogeneously hyperintense on T2 weighted images. Some of these tumors have a central hypointense region giving a “target sign” type appearance on T2 weighted images [22]. However, definitive diagnosis requires a biopsy.

Grossly, plexiform neurofibromas are large lesions that affect large segments of a nerve and contort it into its characteristic appearance of “bag of worms.” Microscopically, it consists of a tortuous mass of enlarged nerve branches which are seen in various planes of cut section. Early stages are characterized by expansion of the endoneurium by myxoid ground substance. With continued growth, spillage of lesional cells occur creating a backdrop of neurofibromatous tissue characterized by interlacing bundles of elongated cells with wavy nuclei intimately associated with wire-like strands of collagen that is separated by small to moderate amounts of mucoid material. The cellular components of neurofibroma consist of varying proportions of Schwann cells, fibroblasts, and peripheral perineural cells with scattered mast cells, lymphocytes, and rare xanthoma cells. Lesions with increased cellularity, atypia, or mitotic figures are at an increased risk for malignant transformation [35].

Currently, there are no specific guidelines for management of PNF involving PH. The most common cause of early death in NF-1 patients is malignant peripheral nerve sheath tumors (MPNST) which most often occur in preexisting PNFs. MPNST have a poor prognosis as they do not respond well to chemotherapy or radiation therapy [36]. Though it was reported that the lifetime risk of malignant transformation to a MPST is 7–13%, the actual transformation rate for intra-abdominal PNFs has not been well described [37]. Given the malignant potential of PNFs, a complete resection is often recommended. This can be achieved in many of the superficially located tumors but not always feasible for tumors located in PH. It is unknown if, extensive resection of deeply situated PNFs is beneficial due to the infiltrating nature and high rate of tumor regrowth [38, 39].

Our findings suggest that in adult patients with NF-1 who have PNF involving PH most of the times the tumor is unresectable on imaging. Even if the tumor appears resectable on imaging, intraoperatively these tumors are found to be unresectable due to intrahepatic extension and extension along the celiac axis. Biopsies can be taken in such instances to rule out transformation. However, it remains unknown if aborted resection with intraoperative biopsies offers any advantage over serial follow-up with MRIs which can help detect transformation as well. Therefore, given the high incidence of intraoperative unresectability in apparently resectable PNF of PH in patients with NF-1, surveillance alone may be a reasonable alternative. Conversely, sporadic PNFs which occur in the absence of NF-1 appear to be more amenable to resection. It can be speculated that the tumors are less extensive in patients with sporadic PNFs compared to those with NF-1 syndrome. PNFs occur as isolated tumors in sporadic cases compared to multiple tumors in those with NF-1. Patients with NF-1 are also at risk for several other malignancies which can prove to be fatal before PNF.

Given the slow growing nature of PNF in general, unknown rate of transformation to malignancy, and high rate of unresectability at exploration, consideration should be given to surveillance alone to assess for growth or development of symptoms. Supporting this approach, Lee et al. reported a case of PNF which infiltrated the lesser sac and hepatic hilum, causing portal hypertension. This patient was treated symptomatically with beta blockers and followed with serial imaging [4]. Delgado et al. have the largest reported experience with PNF of PH. In their series of 5 patients (age < 18 years), all patients were managed conservatively. These lesions remained stable over long-term follow-up (max 8.8 years) [22]. There were no mortalities due to malignant transformation although follow-up is still limited.

In a nice review on malignant peripheral nerve sheath tumors (MPNST), James et al. highlighted the utility of combining PET/CT with CT and MRI to assess for malignant changes [37]. These authors suggested using a SUV max cut-off of 6.1 g/ml (sensitivity 94% and specificity 91%) to differentiate between MPNST and benign nerve sheath tumors [37]. However, none of the studies in the current review used PET/CT for follow-up or surgical decision making. In the opinion of the authors and based on the review of James et al. PET/CT should be part of the radiologic evaluation if surveillance is chosen.

The current study is not without limitations. PNF of PH is a rare condition in general which speaks for the limited number of studies identified. Most of the studies are isolated case reports. An extensive forward search of several databases (PubMed, Scopus, and Web of Science) and backward search from the references of the relevant studies was performed to identify all relevant studies. There are no reports of long-term survival in patients with PNF of PH. This also makes it challenging to assess the risk of malignant transformation at a later time point. Despite the limitations this is the first systematic review focusing on the management of PNF involving the PH

In conclusion, PNFs of the PH are challenging neoplasms. When found in the setting of NF-1 these tumors are often unresectable on imaging. A high incidence of unresectability can be expected at the time of exploration. Given the low or unknown rate of transformation and high incidence of unresectability, surveillance alone can be offered to a subset of patients. The data summarized in the current study can be used to counsel patients at the time of informed consent. If exploration is attempted and unresectability is noted intraoperatively, multiple biopsies can be performed prior to aborting to confirm the diagnosis and rule out transformation. If surveillance is chosen then PET/CT should be combined with CT and MRI to get the most information regarding the biologic behavior of these tumors. Sporadic PNFs of the PH are more likely to be amenable to complete resection. Multidisciplinary management of these tumors should be pursued. We hope that the current study will encourage more authors to report their findings with PNF involving the PH and stimulate further research on these tumors. More studies are needed to evaluate the risk of transformation in these tumors. Given the limited data, a lifelong close follow-up is still recommended. Surveillance alone should be weighed against high incidence of unresectability prior to embarking on surgical management.

## Figures and Tables

**Table 1:** Summary of case reports of management of PNF involving Porta Hepatis in adults.

S. number	Author	Year	Age (Y)	Gender	NF-1	Clinical presentation	MRI/CT if MRI not available	Treatment approach	Path	Follow-up
(1)	Lee et al.	2016	49	M	Yes	Asymptomatic, incidental findings of portal hypertension on EGD and US	Hypointense T1 lesion and low attenuating mass-like lesion with weak enhancement on T2 images	Medical management with beta-blockers for portal hypertension. Mass thought to be unresectable due to extension into hepatic hilum and lesser omentum	-	-

(2)	Poon et al.	2008	40	M	No	4-5-year history of intermittent upper abdominal pain with nausea and vomiting	T1 hypointense and in homogenously T2 hyperintense	Tumor resected en-bloc	Neurofibroma	1 year

(3)	Ghalib et al.	1995	30	F	Yes	Intermittent right upper quadrant pain	Low attenuating mass encasing the left portal vein, extending into the liver, extending into the gastrohepatic ligament, encasement of hepatic artery up to celiac axis	Exploratory laparotomy. Mass found to be unresectable	Plexiform neurofibroma	-

(4)	Rastogi	2008	35	M	Yes	6-month history of vague abdominal pain	Multiple hypoattenuating masses in the liver, porta hepatis, peripancreatic region and retroperitoneum	Nonsurgical management	-	4 months

(5)	Malagari et al.	2001	24	M	Yes	Asymptomatic, incidentally found on US	7 cm well defined lesion in left hepatic lobe extending into porta hepatis and encasing the hepatic artery and celiac trunk	Exploratory laparotomy. Mass found to be unresectable	Plexiform neurofibroma	-

(6)	Hoshimoto et al.	2009	24	F	Yes	Intermittent abdominal pain	T2 hyperintense tumor involving hepatoduodenal ligament and hepatic hilum, extending along intrahepatic Glisson's sheath	The tumor was resected, leaving behind the intrahepatic extension	Plexiform neurofibroma	3 years

(7)	Ji et al.	2017	54	M	No	3-month history of abdominal pain and weight loss of 3 months	3.6 × 1.7 cm homogenous low-attenuation mass at the porta hepatis with irregular infiltrative margins, encasing and spreading along hepatic artery	Tumor resected; tumor was gradually peeled off from the hepatic artery along the arterial sheath	Plexiform neurofibroma	18 months

(8)	Gallego et al.	1998	50	M	Unknown	Unknown symptoms. Isolated neurofibromas in the liver, mediastinum, celiac axis and mesentery	Anomalous mesenteric and retroperitoneal tissue extending through hepatoduodenal ligament in to interhepatic periportal spaces. T1 hypointense and T2 hyperintense	Nonsurgical management	Plexiform neurofibroma	2 years

(9)	Rodríguez et al.	1993	24	M	Yes	Vague abdominal complaints	T1 hypointense and T2 hyperintense. Well defined mass around the porta hepatis and its peripheral branches.	Nonsurgical management		5 months

(10)	Chen et al.	1991	18	M	Yes	Presented with PNF of the skull. Liver PNF incidental	Retroperitoneal neurofibroma with extension into the liver along the portal vein	Nonsurgical management	-	3 months
